# Aerobic exercise intensity does not affect the anabolic signaling following resistance exercise in endurance athletes

**DOI:** 10.1038/s41598-021-90274-8

**Published:** 2021-05-24

**Authors:** T. W. Jones, L. Eddens, J. Kupusarevic, D. C. M. Simoes, M. J. W. Furber, K. A. van Someren, G. Howatson

**Affiliations:** 1 Department of Sport Exercise and Rehabilitation, Northumbria University, Newcastle-upon-Tyne, NE1 8ST UK; 2 Population Health Sciences Institute, Newcastle University, Newcastle-upon-Tyne, UK; 3 Department of Psychology and Sports Science, University of Hertfordshire, Hatfield, UK; 4 Sports Lab North West, Letterkenny Institute of Technology, County Donegal, Ireland; 5 Water Research Group, North West University, Potchefstroom, South Africa

**Keywords:** Physiology, Biomarkers

## Abstract

This study examined whether intensity of endurance stimulus within a concurrent training paradigm influenced the phosphorylation of signaling proteins associated with the mTOR and AMPK networks. Eight male cyclists completed (1) resistance exercise (RES), 6 × 8 squats at 80% 1-RM; (2) resistance exercise and moderate intensity cycling of 40 min at 65% V̇O_2peak_, (RES + MIC); (3) resistance exercise and high intensity interval cycling of 40 min with 6 alternating 3 min intervals of 85 and 45% V̇O_2peak_ (RES + HIIC), in a cross-over design. Muscle biopsies were collected at rest and 3 h post-RES. There was a main effect of condition for mTOR^S2448^ (*p* = 0.043), with a greater response in the RES + MIC relative to RES condition (*p* = 0.033). There was a main effect of condition for AMPKα2^T172^ (*p* = 0.041), with a greater response in RES + MIC, relative to both RES + HIIC (*p* = 0.026) and RES (*p* = 0.046). There were no other condition effects for the remaining protein kinases assessed (*p* > 0.05). These data do not support a molecular interference effect in cyclists under controlled conditions. There was no intensity-dependent regulation of AMPK, nor differential activation of anabolism with the manipulation of endurance exercise intensity.

## Introduction

The ‘interference effect’ describes attenuated strength development within a concurrent strength and endurance training paradigm, in comparison to that following isolated resistance training^1^. Following the seminal work of Hickson^1^ subsequent research has indicated combining strength and endurance training within the same training regimen can result in attenuated strength responses^2,3^. This conflict between opposing sides of the training adaptation continuum can be troublesome for elite and recreational athletes alike.

Previous efforts to explore mechanisms for the interference effect have focused on neuromuscular^4^ and endocrine^3,5^ variables. More recently, research efforts have focused on molecular growth-related signalling^6–8^, and have yielded equivocal findings with little data providing support for a molecular interference effect^9,10^. The majority of research examining an acute interference effect has focused on studying concurrent stimuli *vs.* resistance stimuli in isolation^7,11^, while others have manipulated the concurrent stimuli through an acute training variable, such as exercise sequence^8,10^. Given the variations in endurance training stimuli (ranging from high intensity short intervals to longer duration continuous exercise) there is limited research addressing the role of endurance exercise intensity and how this might be implicated in the purported acute interference effect^12^.

The observed interference effect has been proposed to occur due to a molecular interference, where anabolic signaling and thus myofibrillar protein synthesis (MPS) is impeded by the cellular pathway that regulates energy production for endurance activity^13^. Activating mTORC1 is a pertinent outcome of resistance exercise, owing to its function as a principal mediator of skeletal muscle remodelling^14^. Similarly, the phosphorylation of the protein AMPK is of key importance to the endurance adaptation training process, due to its function of monitoring the energy status of the muscle and initiating aerobic adaptive responses^15^, although recent evidence indicated this might not be the case in endurance trained males^16^. The logic dictates that these two pathways prove antagonistic to one another, given that a role of AMPK is to reduce energy-consuming anabolic processes within the cell^17^. Despite this, there is evidence to the contrary^6^.

Exercise intensity is a key training variable and given that endurance activity is purported to be antagonistic to an early growth response, it would seem logical that a greater endurance exercise load might exacerbate the issue. Experimentally, Rose et al.^18^ observed greater phosphorylation of AMPK following higher intensity cycling exercise (85% V̇O_2peak_) *vs.* lower intensity exercise (35% V̇O_2peak_), supporting the intensity-dependent regulation of AMPK. High intensity interval training can offer adaptations consistent, if not superior, to that of traditional endurance training, with regards to aerobic capacity^19^ and is therefore an appealing training modality. Although, this may be dependent on training status, as previously stated recent work indicates that AMPK may not mediate adaptations in endurance trained males^16^.

If endurance stimuli can impede anabolic signaling processes, it is suggested that endurance training status could be of great relevance. There is strong rationale for trained endurance athletes to undertake resistance training to support athletic performance^20^. Training status has been suggested to modify the early molecular signaling responses of the AMPK and mTOR networks to opposing exercise stimuli, with an attenuated response amongst trained phenotypes and a generic molecular footprint in untrained cohorts^21^. This suggests that untrained individuals to be a poor vehicle to explore the molecular basis of an interference effect. Further, data presented by Coffey et al.^21^ suggested trained athletes are more susceptible to an interference effect. Despite this, there are no data concerning the role of endurance exercise intensity in providing a molecular interference amongst trained endurance athletes. Observation of the molecular response in individuals with this concurrent training status i.e., endurance-trained but relatively strength-naïve, might prove valuable to better understanding the potential to induce a molecular interference.

The aims of this study were twofold. Firstly, to examine whether combining strength and endurance training (independent of intensity) results in the inhibition of anabolic signaling proteins, relative to strength stimuli performed in isolation. Secondly, to observe whether the intensity of the endurance stimuli influences the phosphorylation of signaling proteins associated with the mTOR and AMPK networks. These aims were addressed in a sample of endurance trained, yet strength training naïve cyclists, under more rigorous dietary control than previous work investigating concurrent training and signaling. It was hypothesized that greater phosphorylation of the mTOR and AMPK networks would be observed following more intense endurance stimuli.

## Results

### Physiological response

Blood lactate concentration was greater in the HIIC condition compared with MIC following the endurance exercise stimulus (Table 1), with a condition (*F*_[1,7]_ = 8.264, *p* = 0.024), time (*F*_[1,7]_ = 14.170, *p* = 0.007), and interaction effect observed (*F*_[1,7]_ = 7.608, *p* = 0.028). The HR responses during the different endurance exercise stimuli are listed in Table 1. Maximum HR was greater in the HIIC condition (*p* = 0.001), while average HR across the work- and duration-matched protocols was not different (*p* > 0.05). The time spent in HR zones did not differ between the HIIC and MIC conditions (*p* > 0.05; Table 2).

**Table 1:** Physiological response to the MIC and work-matched HIIC protocols.

Condition	End [La] (mmol·L^−1^)	End + 5 min [La] (mmol·L^−1^)	Max. HR (%max)	Av. HR (%max)
MIC	2.49 ± 1.41	2.04 ± 0.82	91.8 ± 4.3	85.1 ± 4.1
HIIC	6.08 ± 3.83^‡^	4.46 ± 2.66	96.9 ± 3.1^‡^	85.9 ± 3.1

Values presented as mean ± SD. *MIC* moderate intensity cycling; *HIIC* high intensity interval cycling; [*La*] blood lactate concentration; *HR* heart rate; *max*. maximum; *av*. Average.

^‡^Significantly greater than MIC (*p* < 0.05).

**Table 2:** Training load quantification of the MIC and work-matched HIIC protocols.

Condition	Zone 1 (%)	Zone 2 (%)	Zone 3 (%)	Zone 4 (%)	Zone 5 (%)
MIC	1.1 ± 2.1	20.8 ± 19.6	36.7 ± 21.4	30.2 ± 23.9	11.2 ± 25.2
HIIC	0.9 ± 1.2	24.8 ± 16.3	21.0 ± 5.8	31.9 ± 4.5	21.4 ± 17.0

Values presented as mean ± SD. *MIC* moderate intensity cycling; *HIIC* high intensity interval cycling; *zone* (%) % of session in specified heart rate zone. Zone 1: 50–59% of HR_peak_, Zone 2: 60–69% of HR_peak_, Zone 3: 70–79% of HR_peak_, Zone 4: 80–89% of HR_peak_ and Zone 5: ≥ 90% of HR_peak_.

### Signaling response

#### AMPK pathway

**Figure 1:**
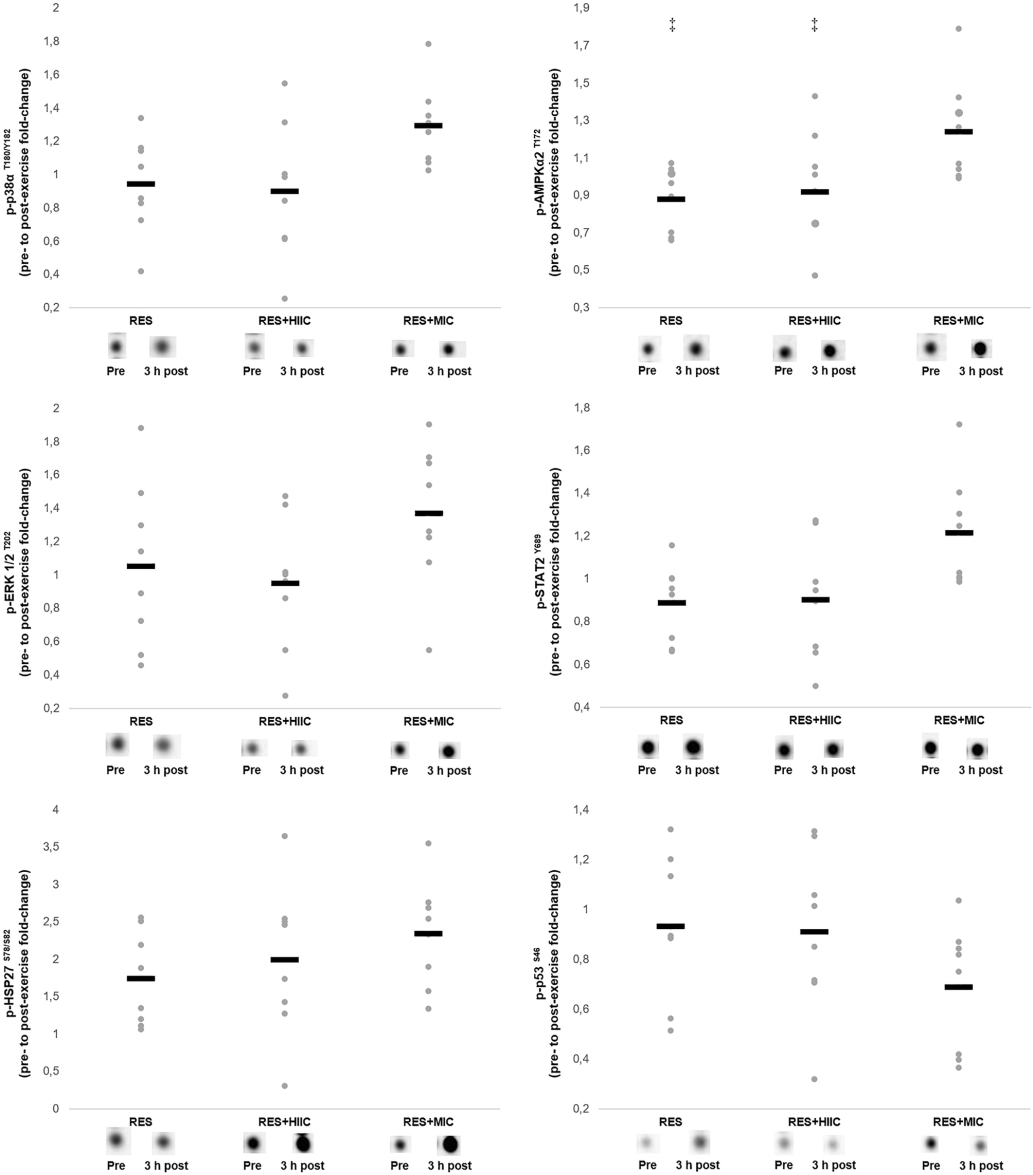
Individual and mean responses in phosphorylation of the AMPK signaling pathway in the RES, RES + MIC, and RES + HIIC conditions including representative images. Grey dots represent individual responses and black lines represent the mean. ‡, significantly different from RES + MIC condition (*p* < 0.05). In all instances all samples from 8 participants were available for analysis.

The signaling response of the protein kinases associated with the endurance pathway are presented in Fig. 1 including representative images. There was a main effect of condition for the phosphorylation of AMPKα2^T172^ (*F*_[2,14]_ = 4.046, *p* = 0.041), with a greater response in RES + MIC, relative to both RES + HIIC (*p* = 0.026) and RES (*p* = 0.046). No other effects of condition were observed for the protein kinases associated with the endurance pathway. Representative images of the mTOR and AMPK networks are presented as a Supplementary Figure.

#### mTOR pathway

**Figure 2:**
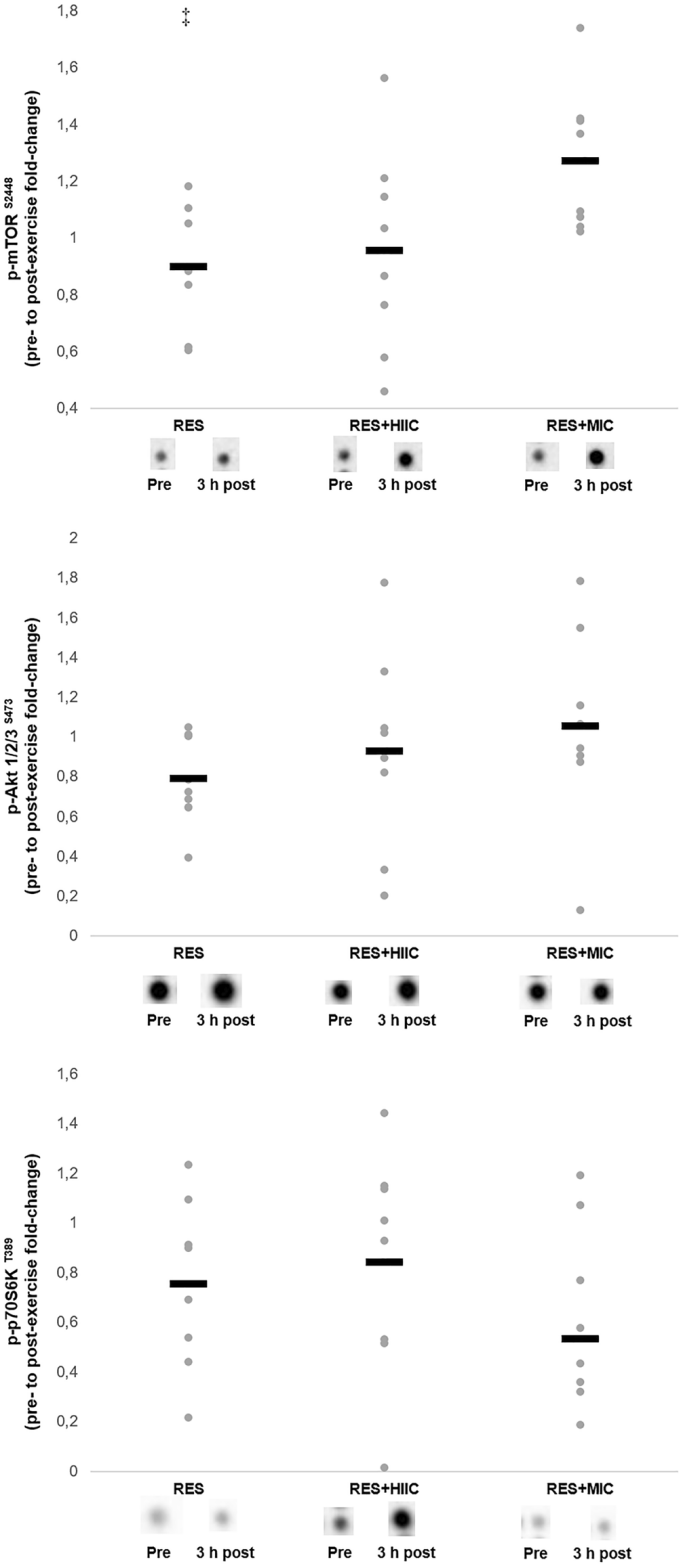
Individual and mean responses in phosphorylation of the mTOR signaling pathway in the RES, RES + MIC, and RES + HIIC conditions including representative images. Grey dots represent individual responses and black lines represent the mean. ‡, significantly different from RES + MIC condition (*p* < 0.05). In all instances all samples from 8 participants were available for analysis.

The signaling response of the protein kinases associated with the strength pathway are presented in Fig. 2 including representative images. There was a main effect of condition for the phosphorylation of mTOR^S2448^ (*F*_[2,14]_ = 3.963, *p* = 0.043), with a greater response in the RES + MIC relative to RES condition (*p* = 0.033). There was no main effect for condition for either Akt1/2/3^S473^ or p70S6K^T389^ phosphorylation (*p* > 0.05).

## Discussion

The present study sought to determine whether a concurrent exercise stimulus (independent of intensity) would result in an inhibition of anabolic signaling proteins, relative to a strength stimulus performed in isolation. Further, this work examined the influence of intensity of the endurance stimuli on the presence or magnitude of a molecular interference effect. These aims were in the context of an endurance-trained, but strength-training naive phenotype under rigorous dietary controls. The major findings were that 1) despite differential AMPK and mTOR signaling between conditions, this was not suggestive of a molecular interference effect i.e., an antagonistic relationship; 2) despite differential activation status of the AMPK and mTOR signaling cascades, this did not support the hypothesis of an intensity-dependent regulation of AMPK.

The majority of research which has investigated molecular interference has focused on studying concurrent stimuli *vs.* resistance stimuli in isolation amongst recreationally active cohorts, using the activation status of the mTOR signaling cascade as a reference for the early response of the anabolic process^7,11,22^. This line of investigation has ultimately provided a lack of support for the theory of a molecular interference effect. Some investigations have reported no difference in the activation status of mTOR and AMPK signaling networks following the manipulation of exercise stimuli^7,8^, however there are also studies that reported differing activation of said networks following combined strength and endurance exercise and strength training alone^6,11,23^.

Apro et al.^6^ reported elevated AMPK phosphorylation in the concurrent exercise condition, relative to a resistance only stimulus, without a subsequent inhibition of mTOR activation status in the concurrent exercise condition. Separate work has reported elevated mTOR and p70S6K activation status following endurance-resistance, compared with resistance only exercise^11^. Similarly, elevated mTOR phosphorylation has been observed in the concurrent condition, relative to resistance exercise^23^. Collectively, this existing literature counter the existence of a molecular interference by failing to demonstrate an antagonistic relationship between mTOR and AMPK signaling networks and elevated mTOR network activity with the removal of endurance stimuli from concurrent exercise. The findings of the present study are consistent, in part, with the aforementioned literature. Firstly, the elevated phosphorylation of AMPK in the RES + MIC *vs.* RES conditions was not observed in conjunction with an inhibition of mTOR activation status in the RES + MIC condition. Secondly, mTOR phosphorylation was upregulated in the RES + MIC *vs.* RES conditions. Hence, while the addition of an endurance stimulus in the RES + MIC condition was sufficient to upregulate AMPK activation status, relative to resistance exercise in isolation, this was consistent with the trend for mTOR phosphorylation and counters the principle of a molecular interference effect.

Exercise intensity is a key training variable, and if endurance activity is purported to be antagonistic to hypertrophic responses, it would seem logical that a greater endurance exercise intensity might exacerbate the issue. This logic has received some investigation in the context of recreationally-active individuals^12^, with resistance exercise in isolation being compared with work and duration-matched concurrent models, incorporating either moderate or high intensity exercise. In the study of Fyfe et al.^12^ AMPK phosphorylation was similarly upregulated across all conditions, while mTOR activation status was only elevated in the high intensity concurrent condition. In addition, despite p70S6K activation status increasing following each of the conditions, there was no difference in the response between conditions^12^. Hence, these data from previous research indicate that concurrent exercise with a high intensity endurance component would be preferential compared to resistance exercise in isolation or a moderate-intensity concurrent stimulus, in creating an anabolic environment. Elevated mTOR signaling has previously been observed following intensive cycling exercise in untrained individuals^24^, in agreement with the premise of a generic molecular footprint following exercise that the individual is unaccustomed to^25^. If the recreationally active cohort from the work of Fyfe et al.^26^ were less accustomed to high intensity endurance activity, relative to moderate intensity stimuli, this might help to explain the observed mTOR phosphorylation responses. Further, the data fail to support the intensity-dependent regulation of AMPK, which has also been refuted in a running exercise model^27^.

The work of Fyfe et al.^26^ offers data consistent with the key findings of this study, in respect to a lack of evidence for both a molecular interference effect and the intensity-dependent regulation of AMPK. However, an inconsistency was that the greatest mTOR activation status was observed with a higher intensity of endurance exercise; the data from this study suggest that the moderate intensity concurrent stimulus would be preferential. Although Fyfe et al.^26^ observed greater mTOR activation with a higher intensity of endurance exercise following an 8-week training intervention, rather than in an acute paradigm. Regardless of which concurrent stimulus provided a superior stimulus of the anabolic process both contradict the theory of a molecular interference effect, in which the resistance only stimulus would afford an enhanced strength response. Whilst unexpected, this finding is not unique; others have reported augmented mTOR activation status following concurrent exercise *vs.* resistance exercise in isolation^11^.

Numerous investigations concerning the acute interference effect have utilized an endurance followed by resistance exercise model as a concurrent stimulus^6,11,12,21^. This is to examine the potential inhibitory effect of prior endurance exercise on the anabolic stimulus. However, A meta-analysis on the influence of concurrent strength and endurance exercise order^28^ indicates a beneficial effect of a resistance followed by endurance exercise for lower-body strength adaptation across a short-term concurrent training programme, when compared with endurance followed by strength training. This would suggest that strength prior to endurance stimulus is an appropriate model to examine the interference effect; an exercise sequence which is beneficial for strength adaptation and thus a more ecologically valid model.

The exercise stimuli used in this study resulted in a generally small response in the phosphorylation of protein targets compared with some of the existing literature. This study observed peak magnitudes of ~ 1.5-fold increase in the phosphorylation of targets, except for HSP27 (~ 2.5-fold). Previous research reported substantial perturbations to downstream targets of the mTOR signaling network in response to exercise; a 14-fold^7^, 12-fold^6^, and 16-fold^29^ increase in p70S6K activation status at residue Thr^389^. However, there are data which highlight the variability in quantifying signaling responses associated with the mTOR and AMPK signaling cascades, reflected by observations of much smaller magnitudes of upregulation or even decreased activation status in protein targets; a 1.8-fold increase in p70S6K^T389^ activation status^11^; a decrease in Akt^S473^ phosphorylation^23^; a decrease in AMPK^T172^ phosphorylation^7^. Hence, there is a lack of agreement in the activation status of key targets within the signaling cascades. Furthermore, it may be speculated that the fact the population studied here were strength training naïve contributed to the lack of phosphorylation of the mTOR network, or perhaps that the resistance exercise stimulus was insufficient.

Where discrepancies do present in the magnitude of signaling responses between this work and existing literature, this might be explained by the numerous methodological variables encountered with a concurrent exercise model. Training status in an important consideration when interpreting the signaling responses to exercise stimulus, as previous work has indicated that signaling responses are attenuated in highly trained individuals^25^.Given the endurance training status of the participants in this study, an attenuated response in the phosphorylation of targets associated with the AMPK signaling cascade could have been anticipated. However, the response in anabolic signaling was somewhat more surprising, with limited phosphorylation of mTOR and downstream targets. Nutrition is another non-training variable capable of modulating the molecular response to an exercise stimulus, with the provision of amino acids inducing a stimulatory effect on the growth-associated signaling network^30^. As such, great effort was employed to control diet in this work. Previous work investigating concurrent training and the mTOR-AMPK axis has not employed rigorous dietary controls. Much of the previous research has asked participants to record and duplicate food intake^6,29^, or has utilized a fasted model of exercise^7,9^. In line with other models^8,11,12^, this work provided a small amount of protein prior to exercise (0.1 g·kg^−1^·d^−1^ protein). This inconsistency in method could increase the magnitude of mTOR signaling, and hence fail to explain the smaller magnitude of change observed in comparison to some previous research^6,7,29^. However, in the present study rigorous standardization of nutritional intake 24-h prior to exercise and a robust within-subject repeated measures design was employed. As such, it is reasonable to suggest that any meaningful differences in phosphorylation of the mTOR and AMPK networks would have been detected.

Other training programme variables might also act to modulate the adaptive response to exercise stimuli. For example, an exercise mode of running *vs.* cycling^2^, an increase in endurance session frequency^3^, and reduced recovery between exercise modes^31^ have been reported to increase the likelihood of interference. In the current study, the decision was made to better represent the applied scenario and the nature of athletic training. As such, this work used a strength exercise model to better mimic the real-life training setting. The use of a leg press machine or dynamometer is common within the body of literature^9,21,23^, despite the fact that the use of fixed resistance equipment exhibits questionable ecological validity. Instead, the current work employed a back-squat exercise to stimulate the *quadriceps*. This exercise has been reported to activate the *vastus lateralis* similarly compared with alternative resistance training exercises^32^. Therefore, while inconsistent with some of the previous literature, the use of this exercise is unlikely to explain the moderate activity of the molecular targets measured.

The present study in not without limitations. Firstly, although a robust repeated measures crossover design was employed the sample size is low, although greater than some comparable previous research^8^. Time in HR zones 1–5 was similar between MIC and HIIC protocols. As such, from a cardiovascular perspective it could be suggested that physiological stress was similar between conditions. However, this lack of statistical significance is largely attributable to large inter-individual variation in the data. Furthermore, blood lactate concentrations were greater following HIIC, and it is likely that HIIC induced greater metabolic stress than MIC. It is also important to note that due to the experimental protocols employed here the RES condition involved a 45 min period with no exercise stimulus prior to the post exercise biopsies, whereas RES + MIC and RES + HIIC involved 45 min of cycling. It is possible that this longer “recovery” period could have contributed to lesser mTOR and AMPK signaling in the RES condition. Furthermore, the timing of the post exercise biopsy (3 h post endurance stimulus) may have influenced any differential signaling responses between conditions and is it possible that a longer post exercise observational period would have been beneficial. However, previous work examining the mTOR-AMPK axis in an acute concurrent training paradigm has employed a post exercise observational period of 3 h^6–8^.

In conclusion, these data do not support an acute molecular interference effect in a trained endurance cohort. The data also fail to support the intensity-dependent regulation of AMPK, when comparing a work and duration-matched moderate and high intensity concurrent exercise stimulus. Finally, the findings add to the growing body of literature, suggestive of mTORC1 and AMPK to be poor correlates to investigate the mechanism explaining concurrent interference, particularly in an acute exercise paradigm. These data suggest that endurance athletes need not be concerned with the intensity of their endurance session (moderate *vs.* high intensity) affecting their strength adaptation, when the two exercise modes are performed in close proximity to one another.

## Methods

### Design

The study utilized a within-subject, repeated measures design. Following two preliminary trials for familiarization to procedures and collection of subject characteristics, participants attended the laboratory on three further occasions. Participants were randomized to complete either 1) resistance exercise (RES) only; 2) resistance exercise followed by moderate intensity cycling (RES + MIC); 3) resistance exercise followed by work- and duration-matched high intensity interval cycling (RES + HIIC). The randomization of trial orders was balanced and conducted via an online randomization platform (www.randomization.com). Visits were separated by ~ 1 wk (range: 6–14 days) and participants were deemed fit for testing if they could confirm that they were free from medications/vitamin supplementation and had refrained from external exercise, caffeine and alcohol for 24 h. Participants were asked to maintain habitual diet and exercise practices throughout the duration of the study.

Preliminary data were collected for stature, body mass, and V̇O_2peak_. Preliminary visits also involved coaching of the back squat exercise and 5-RM assessment. V̇O_2peak_ and 5-RM data were used to prescribe relative exercise intensities for the three experimental trials. The experimental trials required participants to complete RES only (6 × 8 squat repetitions at 80% predicted 1-RM), or an identical stimulus, followed by either MIC (40 min cycling at 65% V̇O_2peak_) or HIIC (40 min cycling with 3 min intervals of 85 and 45% V̇O_2peak_).

### Participants

Eight males (age 32 ± 5 years; stature 1.79 ± 0.04 m; mass 70.6 ± 7.0 kg; V̇O_2peak_ 55.4 ± 7.1 ml∙kg^−1^∙min^−1^; power output at V̇O_2peak_ 358 ± 28 W) volunteered to take part in the study. All participants were trained endurance cyclists with 4 ± 3 years competitive cycling experience, were currently performing 4 ± 1 cycling training sessions·wk^−1^ and were regularly competing (at least a Category 3 British Cycling license holder or an estimated 16.1 km time trial of ≤ 23 min). Volunteers had to possess an endurance training history of > 1 year, with no apparent contraindications to the study and participants had no resistance training history for ≥ 6 months prior to enrolment. All participants were non-smokers and not permitted to consume nutritional supplements. Forty participants were phone screened, 8 of these individuals both met the aforementioned criteria and were available to participate in the study. After being informed of the potential benefits and risks and completing a questionnaire to assess for eligibility and contraindications to the study, participants volunteered to take part in the research by providing written, informed consent. All documentation and procedures were approved by the Northumbria University Research Ethics Committee, in accordance with the Declaration of Helsinki.

### Procedures

#### Preliminary testing

Two preliminary visits were undertaken at least 1 wk prior to the three experimental trials. At visit 1, data were collected for height and body mass (Seca 704 r, Seca., Hamburg, Germany), followed by an assessment of V̇O_2peak_ and initial coaching of the squat exercise. The subsequent preliminary visit was used to further coach the squat exercise and assess 5-RM for the back-squat.

#### Assessment of peak oxygen uptake

In all cases participants fit their own clip in pedals to the cycle ergometer. Participants were instructed to set up the ergometer (saddle and handlebar height and saddle fore/aft position) to mimic their on-road cycling position, settings were recorded and kept consistent across all experimental trials. Participants were also instructed to remain seated throughout assessments and training sessions.

An incremental lactate threshold (LT) assessment was conducted prior to the V̇O_2peak_ test, with the starting intensity selected (range: 125–200 W) to initiate the test below the LT, with subsequent increases in the work rate of 25 W every 4 min. This assessment was terminated with a blood lactate concentration of ≥ 4 mmol·l^−1^ (range: 4–7 stages). After completion of the lactate threshold assessment, a 15 min period of rest was initiated. Participants then cycled at a power output of 200 W using an electro-magnetically braked cycle ergometer (Velotron, RacerMate Inc., Seattle, USA). Power output was subsequently increased by 4 W every 10 s (24 W·min^−1^) until volitional exhaustion. Expired gas and heart rate (HR) were collected throughout the test, with the test being terminated when the participant was unable to maintain the workload. Expired gas data was collected via online breath by breath analysis (Metalyzer 3B, Cortex., Leipzig, Germany) data were averaged across 30 s intervals; V̇O_2peak_ was calculated as the highest 30 s average during the maximal test. Routine quality assurance records demonstrated a laboratory coefficient of variation for V̇O_2_ data of 1.8% in the range of 2.05–3.94 l·min^−1^.

#### Maximal strength testing

Maximal strength was predicted from participants’ 5-RM performance in the parallel high bar barbell back squat exercise, using the following; *Eq. (1) 1-RM* = *100 · rep wt/(48.8* + *53.8 · exp [-0.075 · reps]*^33^ which previously reported good agreement with 1-RM performance in individuals naïve to strength training^34^. It was deemed that a 5-RM assessment would be a safer method of assessment for the participants, given their lack of strength training experience. The squat technique is reported to provide a potent stimulus of the *vastus lateralis*, comparative to that of alternate lower-body strength exercises^32^. The assessments were conducted in line with standardized procedures^20,32^ and supervised by a qualified strength and conditioning coach who ensured correct form and technique. A standardized warm-up, lasting approximately 10 min, was completed prior to testing. This warm-up consisted of shuttle runs, inch worms, lunges, walking Romanian deadlifts, squat rotations, glute bridges, and planks, followed by three sets of back-squat of increasing load (40, 75, and 85% of predicted 1-RM) and decreasing number of repetitions (10, 8, and 6, respectively). The first 5-RM attempt was lifted with a load ~ 5% below the predicted 5-RM. After each successful attempt, the load was increased by 2–5%, with one re-attempt permitted per load. The rest period between each attempt was standardized at 3 min and the last successful set was accepted as the 5-RM score.

#### Resistance exercise stimulus

After performing the same standardized warm up as prior to the 5-RM assessments, participants completed two warm-up sets of the back-squat (10 and 8 repetitions at 40 and 60% of predicted 1-RM, respectively). Consistent with the maximal strength testing, RES was conducted in line with standardized procedures and supervised by a qualified strength coach. Participants completed 6 × 8 repetitions at 80% of predicted 1-RM, with the rest period between each set standardized at 3 min. This session composition is in agreement with the ACSM position stand^35^ and similar session composition has been reported to upregulate protein phosphorylation targets of the mTOR pathway^10,36^. If prescribed, participants commenced the endurance exercise stimulus (described subsequently) within 5 min of completing RES. HR and blood lactate concentrations were not recorded during the resistance exercise stimulus.

#### Endurance exercise stimulus

**Figure 3:**
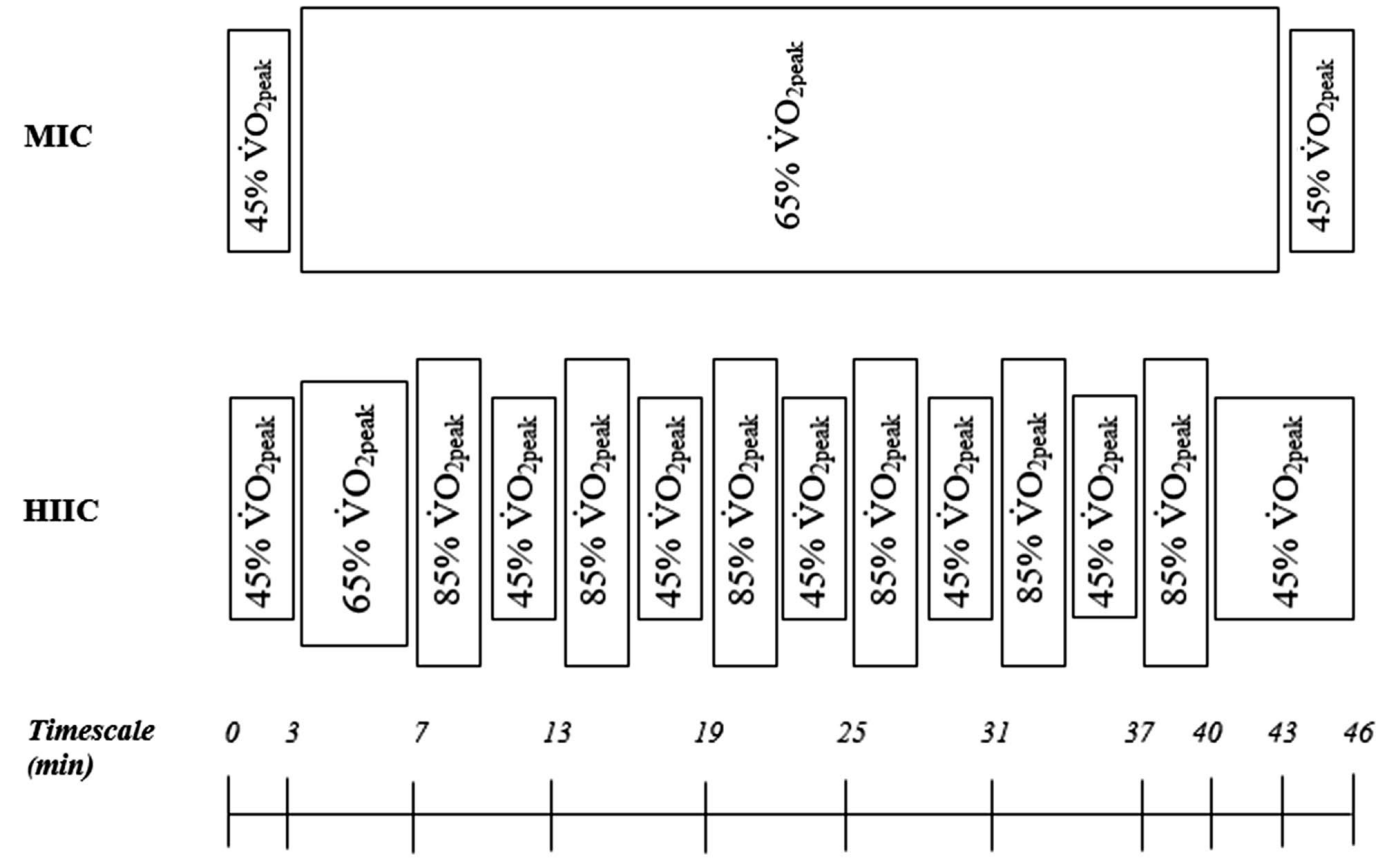
Schematic of the work and duration-matched MIC and HIIC protocols, forming the endurance exercise element of the acute exercise stimulus. *MIC* moderate intensity cycling; *HIIC* high intensity interval cycling.

MIC entailed constant load cycling at a fixed power output (W) equivalent to 65% V̇O_2peak_, while HIIC required participants to perform 3 min intervals at W equivalent to 85 and 45% V̇O_2peak_, using the cycle ergometer. The MIC condition of 65% V̇O_2peak_ provided a standard prescription of endurance exercise intensity of constant duration^7,8,10^. The exercise of 3 min intervals of 85 and 45% V̇O_2peak_ provided a matched high intensity intervention^37^. Further, similar stimuli have previously been used to investigate biochemical responses to endurance activity^27^. Both protocols contained a warm-up and cool down and are presented in Fig. 3. HR was recorded throughout each trial (Polar A300 transmitter, Polar Electro Ltd., Kempele, Finland), while visual feedback of time elapsed, power output, and pedal cadence were made available to participants. Time in HR zones was calculated during both MIC and HIIC protocols, with zones defined as; zone 1: 50–59% of peak HR (HR_peak_), zone 2: 60–69% of HR_peak_, zone 3: 70–79% of HR_peak_, zone 4: 80–89% of HR_peak_ and zone 5: ≥ 90% of HR_peak_.

#### Exercise and dietary controls

For 24 h prior to an experimental trial, participants refrained from structured exercise and consumed a standardized diet. No participants reported performed any strenuous or “heavy” exercise for 72 h prior to the experimental trials. Dietary intake was controlled for 24 h prior to the first experimental trial until the completion of all 3 trials. Daily dietary intake was standardized (6 g·kg^−1^·d^−1^ carbohydrate, 1.3 g·kg^−1^·d^−1^ protein, 0.98 g·kg^−1^·d^−1^ fat), with the evening meal (1900 h) and breakfast meal (0600 h) prior to the visit standardized at 3 g·kg^−1^·d^−1^ carbohydrate, 0.5 g·kg^−1^·d^−1^ protein, 0.3 g·kg^−1^·d^−1^ fat and 1 g·kg^−1^·d^−1^ carbohydrate, 0.1 g·kg^−1^·d^−1^ protein, < 0.01 g·kg^−1^·d^−1^ fat, respectively. Following the breakfast meal participants consumed nothing other than water (ad libitum) until after the final muscle biopsy was performed 3 h post exercise. The diet was sourced from a bespoke menu provider (Soulmatefood Ltd., Lancashire, UK) and designed in line with American College of Sports Medicine (ACSM) recommendations^38^. Food was delivered to participants’ home address and labelled such that four meals were provided daily, to be consumed at specified times, ensuring that food distribution throughout the day was standardized. Food content was analyzed using dietary analysis software (Nutritics v4.108, Nutritics Ltd., Co. Dublin, Ireland), to confirm nutritional content was correct. Prior to experimental procedures participants were asked whether they adhered to the prescribed diet and consumed all the food provided. All participants indicated that they had adhered to the prescribed diet prior to all experimental trials.

#### Muscle tissue sampling

**Figure 4:**
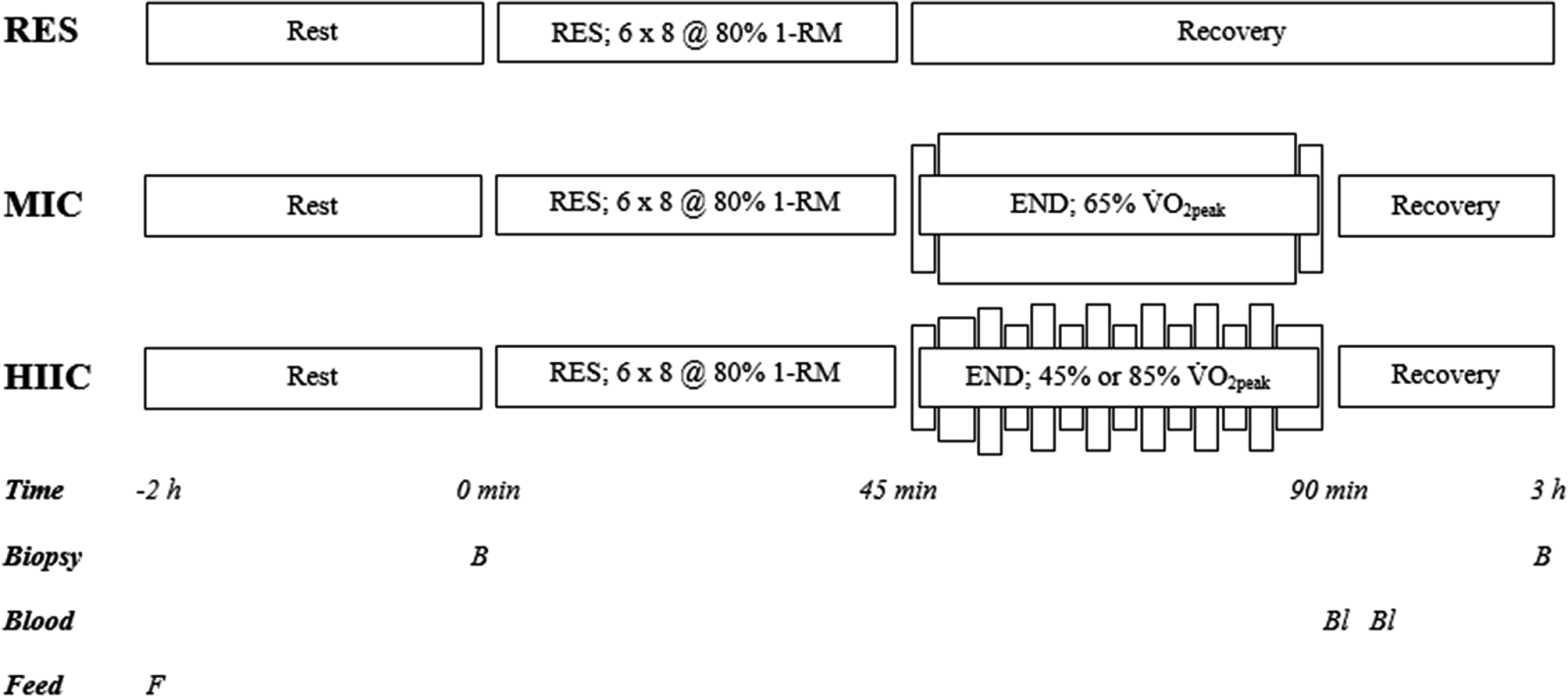
Schematic of the experimental and sampling timeline. *RES* resistance exercise; *END* endurance exercise.

Upon arrival at the laboratory (~ 0730 h), participants were screened for contraindications to the muscle biopsy procedure, before resting in a supine position (10 min). All sessions commenced at the same time of day (± 30 min), to minimize the effects of diurnal variation in molecular responses to the exercise stimuli^39^. Muscle samples were collected from the middle portion on the lateral aspect of the *vastus lateralis* muscle, using the micro-muscle biopsy technique. The site was disinfected with Betadine (Purdue Pharma., Connecticut, USA) and samples were obtained under local anesthesia, with 2 ml of 1% Lidocaine Hydrochloride (Hameln Pharmaceuticals., Gloucester, UK) injected into the subcutaneous tissue of the biopsy site. After confirming that the anesthetic had taken affect (~ 5 min), a 14-gauge co-axial needle was inserted ~ 2 cm into the muscle (beyond the subcutaneous tissue). A disposable biopsy instrument (TSK Stericut Biopsy Needle 14 Gauge, TSK Laboratories, Tochigi, Japan) was subsequently inserted through the co-axial and discharged. A single muscle sample was collected (~ 10–20 mg) and the tissue was immediately frozen in liquid nitrogen, before being stored at − 80 °C until subsequent analysis. If required, a second pass was completed, with the biopsy instrument rotated 180° inside the co-axial needle and a second sample obtained by turning the needle in the opposite direction of the first pass. Biopsies were obtained immediately prior to RES and 3 h after completion of RES, with participants resting in a waiting room for the interval between the end of exercise and the final biopsy. All within-trial biopsies were sampled from the same leg, while between-trial biopsies were sampled from alternate legs. The second sample of the visit was collected ~ 3 cm proximal the first sample, to avoid a down-stream hematoma, which has been observed with the more invasive Bergstrom muscle biopsy technique^40^. A schematic representation of the experimental and sampling timeline is presented in Fig. 4.

#### Blood lactate sampling

Capillary blood samples were collected from the fingertip at the end of, and following, 5 min passive recovery from MIC and HIIC. Twenty µL samples were collected into a capillary tube and analyzed immediately using an automated blood lactate analyzer (Biosen C-Line, EKF Diagnostics., Cardiff, UK). Routine quality assurance records demonstrated a laboratory coefficient of variation for blood lactate measurement of 0.27%, in the range of 2–18 mmol·l^−1^.

### Muscle analysis

All muscle samples were analyzed using a human phospho-kinase array (Proteome Profiler; no. ARY003B, R&D Systems., Minneapolis, USA), as per the manufacturer’s instructions. Briefly, approximately 10 mg of muscle tissue was homogenized in ice-cold lysis buffer. Tissues were homogenized using Omni Rotor Stator Tissue Homogenizer (TH). Cells were lysed in Phosphatase Inhibitor Cocktail PhosphoSTOP (Cat No 04906845001, Roche) in combination with the Complete Protease Inhibitor Cocktail (Roche) buffer according to supplier’s instructions. Samples were rotated end-over-end for 30 min at 4 °C and centrifuged at 13,000 g for 6 min, and the supernatant subsequently collected. Protein concentration was determined using a total protein assay (Pierce BCA Protein Assay; no. 23225, Thermo Scientific., Rockford, USA), with a starting range of 400 µg per array. The nitrocellulose membranes with spotted capture and control antibodies, were blocked with array buffer 1 for 1 h at room temperature on a rocking platform shaker. Cell lysates were then diluted to a final volume of 2 ml with array buffer 1 and membranes rocked in solution overnight at 4 °C. Membranes were subsequently washed to remove unbound proteins and incubated for 2 h at room temperature with the respective antibody solution (diluted detection antibody cocktail A or B). After washing, membranes were incubated for 30 min in a diluted streptavidin horseradish-peroxidase solution and protected from light, while being rocked at room temperature. After being washed again, chemiluminescent detection reagents were spread evenly onto the membranes and incubated for 1 min, before removing excess solution and measuring the amount of bound phosphorylated protein with a 15 min exposure, using a Syngene G:Box XR5 imaging system with GeneSys analysis software (Syngene., Cambridge, UK). All sample time points for each participant were run in parallel.

After imaging, the average signal produced at the duplicate capture spots was quantified for each phosphorylated kinase protein with the ImageJ application (National Institute of Health, USA). In brief, the region of interest on each membrane was measured with the same frame, producing a pixel density for each spot. An inverted value was calculated per protein, with net values calculated by subtracting the inverted background. Finally, a protein ratio value was calculated by taking a ratio of the net value over the reference control, allowing for the relative quantification of phosphorylation between experimental conditions.

### Statistical analysis

Data are presented as mean ± SD, with statistical significance set at p ≤ 0.05 a priori. Sphericity was assumed if Mauchly’s test score returned *p* ≥ 0.05, with Greenhouse-Geiser adjustments made where appropriate. Blood [La] and HR measures were analyzed using a condition (RES + MIC *vs.* RES + HIIC) by time-point (immediately post- and 5 min post-END) repeated measures ANOVA. The difference in the phosphorylation profile of kinases between-trials was analyzed using a one-way ANOVA with repeated measures, comparing condition (RES *vs.* RES + MIC *vs.* RES + HIIC) for the fold-change response in phosphorylation from rest to 3 h post-RES. Significant main effects were further investigated using LSD post-hoc, pair-wise comparisons. All data analysis was performed using statistical software (IBM SPSS 22 for Windows., New York, USA).

## Supplementary Information

Supplementary Information.

## References

[1] Hickson RC (1980). Interference of strength development by simultaneously training for strength and endurance. Eur. J. Appl. Physiol. Occup. Physiol..

[2] Wilson JM (2012). Concurrent training: a meta-analysis examining interference of aerobic and resistance exercises. J. Strength Cond. Res..

[3] Jones TW, Howatson G, Russell M, French DN (2016). Performance and Endocrine Responses to Differing Ratios of Concurrent Strength and Endurance Training. J. Strength Cond. Res..

[4] Jones TW, Howatson G, Russell M, French DN (2013). Performance and neuromuscular adaptations following differing ratios of concurrent strength and endurance training. J. Strength Cond. Res..

[5] Kraemer WJ (1995). Compatibility of high-intensity strength and endurance training on hormonal and skeletal muscle adaptations. J. Appl. Physiol..

[6] Apró W (2015). Resistance exercise-induced S6K1 kinase activity is not inhibited in human skeletal muscle despite prior activation of AMPK by high-intensity interval cycling. Am. J. Physiol. Metab..

[7] Apró W, Wang L, Pontén M, Blomstrand E, Sahlin K (2013). Resistance exercise induced mTORC1 signaling is not impaired by subsequent endurance exercise in human skeletal muscle. Am. J. Physiol. Metab..

[8] Jones TW (2016). Signaling responses after varying sequencing of strength and endurance training in a fed state. Int. J. Sports Physiol. Perform..

[9] Coffey VG (2009). Effect of consecutive repeated sprint and resistance exercise bouts on acute adaptive responses in human skeletal muscle. Am. J. Physiol. Regul. Integr. Comp. Physiol..

[10] Coffey VG, Pilegaard H, Garnham AP, O’Brien BJ, Hawley JA (2009). Consecutive bouts of diverse contractile activity alter acute responses in human skeletal muscle. J. Appl. Physiol..

[11] Lundberg TR, Fernandez-Gonzalo R, Gustafsson T, Tesch PA (2012). Aerobic exercise alters skeletal muscle molecular responses to resistance exercise. Med. Sci. Sport. Exerc..

[12] Fyfe JJ, Bishop DJ, Zacharewicz E, Russell AP, Stepto NK (2016). Concurrent exercise incorporating high-intensity interval or continuous training modulates mTORC1 signaling and microRNA expression in human skeletal muscle. Am. J. Physiol. Integr. Comp. Physiol..

[13] Coffey VG, Hawley JA (2017). Concurrent exercise training: Do opposites distract?. J. Physiol..

[14] Philp A, Hamilton DL, Baar K (2011). Signals mediating skeletal muscle remodeling by resistance exercise: PI3-kinase independent activation of mTORC1. J. Appl. Physiol..

[15] Hardie DG (2004). AMP-activated protein kinase: A key system mediating metabolic responses to exercise. Med. Sci. Sport. Exerc..

[16] McConell GK, Wadley GD, Le Plastrier K, Linden KC (2020). Skeletal muscle AMPK is not activated during 2 h of moderate intensity exercise at ∼65% V ˙ O 2 peak in endurance trained men. J. Physiol..

[17] Jørgensen SB, Richter EA, Wojtaszewski JFP (2006). Role of AMPK in skeletal muscle metabolic regulation and adaptation in relation to exercise. J. Physiol..

[18] Rose AJ, Bisiani B, Vistisen B, Kiens B, Richter EA (2009). Skeletal muscle eEF2 and 4EBP1 phosphorylation during endurance exercise is dependent on intensity and muscle fiber type. Am. J. Physiol. Integr. Comp. Physiol..

[19] Wisløff U (2007). Superior cardiovascular effect of aerobic interval training versus moderate continuous training in heart failure patients. Circulation.

[20] Rønnestad BR, Hansen EA, Raastad T (2010). Effect of heavy strength training on thigh muscle cross-sectional area, performance determinants, and performance in well-trained cyclists. Eur. J. Appl. Physiol..

[21] Carrithers J, Carroll C, Coker R, Sullivan D, Trappe T (2007). Concurrent exercise and muscle protein synthesis: implications for exercise countermeasures in space. Aviat. Space. Environ. Med..

[22] Fernandez-Gonzalo R, Lundberg TR, Tesch PA (2013). Acute molecular responses in untrained and trained muscle subjected to aerobic and resistance exercise training versus resistance training alone. Acta Physiol..

[23] Pugh, J. K., Faulkner, S. H., Jackson, A. P., King, J. A. & Nimmo, M. A. Acute molecular responses to concurrent resistance and high-intensity interval exercise in untrained skeletal muscle. *Physiol. Rep.* **3**, e12364 (2015). 10.14814/phy2.12364 PMC4425969 25902785

[24] Mascher H, Ekblom B, Rooyackers O, Blomstrand E (2011). Enhanced rates of muscle protein synthesis and elevated mTOR signalling following endurance exercise in human subjects. Acta Physiol..

[25] Coffey VG (2006). Early signaling responses to divergent exercise stimuli in skeletal muscle from well-trained humans. FASEB J..

[26] Fyfe, J. J., Bartlett, J. D., Hanson, E. D., Stepto, N. K. & Bishop, D. J. Endurance training intensity does not mediate interference to maximal lower-body strength gain during short-term concurrent training. *Front. Physiol.* **7**, (2016). 10.3389/fphys.2016.00487 PMC5093324 27857692

[27] Bartlett JD (2012). Matched work high-intensity interval and continuous running induce similar increases in PGC-1α mRNA, AMPK, p38, and p53 phosphorylation in human skeletal muscle. J. Appl. Physiol..

[28] Eddens L, van Someren K, Howatson G (2018). The role of intra-session exercise sequence in the interference effect: a systematic review with meta-analysis. Sports Med..

[29] Wang L, Mascher H, Psilander N, Blomstrand E, Sahlin K (2011). Resistance exercise enhances the molecular signaling of mitochondrial biogenesis induced by endurance exercise in human skeletal muscle. J. Appl. Physiol..

[30] Deldicque L, Theisen D, Francaux M (2005). Regulation of mTOR by amino acids and resistance exercise in skeletal muscle. Eur. J. Appl. Physiol..

[31] Robineau J, Babault N, Piscione J, Lacome M, Bigard AX (2016). Specific training effects of concurrent aerobic and strength exercises depend on recovery duration. J. Strength Cond. Res..

[32] Ebben W (2009). Muscle activation during lower body resistance training. Int. J. Sports Med..

[33] Wathan, D. Load Assignment. in *Essentials of Strength Training and Conditioning* (Human Kinetics, 1994).

[34] LeSuer D, McCormick J, Mayhew J, Wasserstein R, Michael D (1997). The accuracy of prediction equations for estimating 1-rm performance in the bench press, squat, and deadlift. J. Strength Cond. Res..

[35] Progression models in resistance training for healthy adults. *Med. Sci. Sport. Exerc.* **41**, 687–708 (2009). 10.1249/MSS.0b013e3181915670 19204579

[36] Parr, E. B. *et al.* Alcohol ingestion impairs maximal post-exercise rates of myofibrillar protein synthesis following a single bout of concurrent training. *PLoS ONE* **9**, e88384 (2014). 10.1371/journal.pone.0088384 PMC3922864 24533082

[37] Seiler S, Tønnessen E (2009). Intervals, thresholds, and long slow distance: the role of intensity and duration in endurance training. Sportscience.

[38] Thomas DT, Erdman KA, Burke LM (2016). Position of the academy of nutrition and dietetics, dietitians of Canada, and the American College of Sports Medicine: Nutrition and athletic performance. J. Acad. Nutr. Diet..

[39] Atkinson G, Reilly T (1996). Circadian variation in sports performance. Sport. Med..

[40] Van Thienen, R., D’Hulst, G., Deldicque, L. & Hespel, P. Biochemical artifacts in experiments involving repeated biopsies in the same muscle. *Physiol. Rep.* **2**, e00286 (2014). 10.14814/phy2.286 PMC4098731 24819751

