# Supplementary figures and images for: Aerobic exercise intensity does not affect the anabolic signaling following resistance exercise in endurance athletes
Source: Sci Rep. 2021 May 24;11:10785. doi: 10.1038/s41598-021-90274-8 (PMC8144549; doi:10.1038/s41598-021-90274-8)

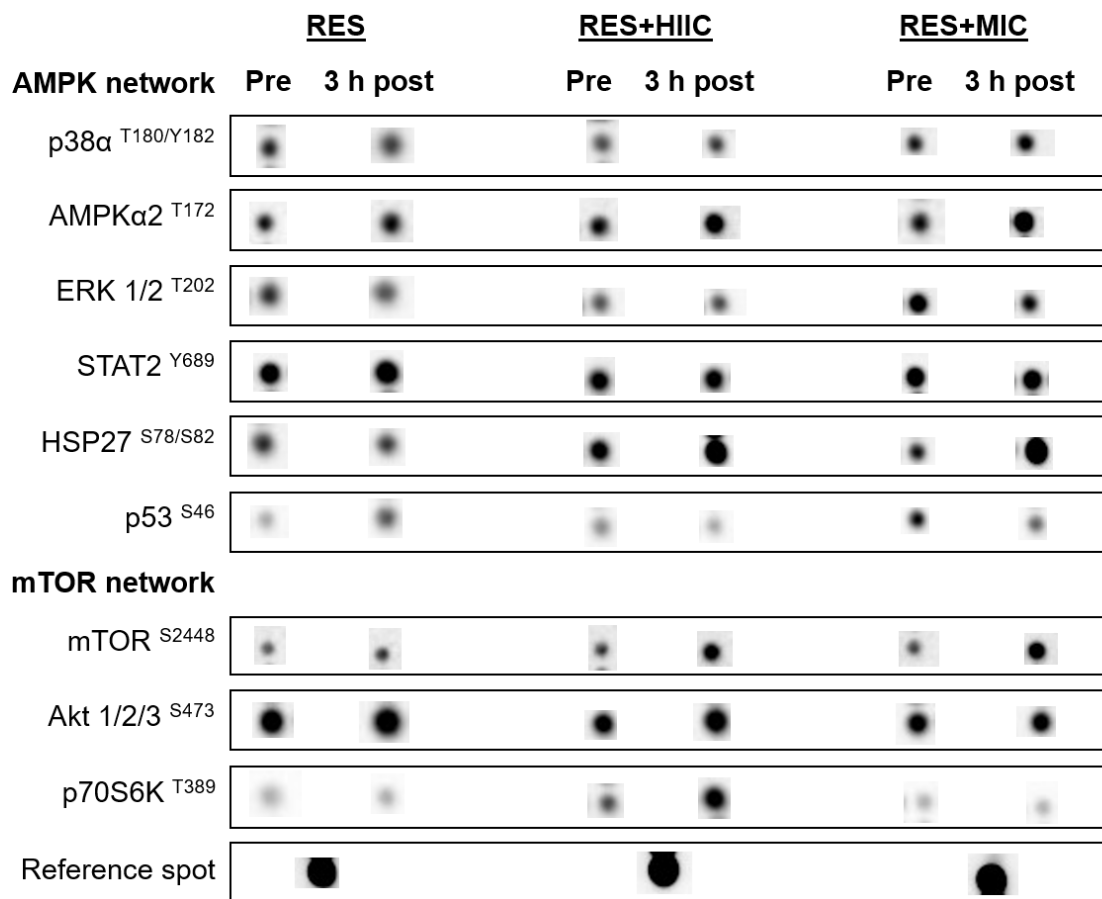

**Supplementary Figure.** Representative images of proteins analyzed.

Supplement: Supplementary file 1 — Supplementary Information. [file 41598_2021_90274_MOESM1_ESM.pdf]
